# Parental reflective functioning and internalizing symptoms predict altruistic prosocial behaviour in children

**DOI:** 10.1111/bjdp.12551

**Published:** 2025-02-17

**Authors:** Daniel McGlade, Helena Rutherford, Eamon McCrory, Nikolaus Steinbeis

**Affiliations:** ^1^ Division of Psychology and Language Sciences University College London London UK; ^2^ Anna Freud London UK; ^3^ Yale Child Study Center, Yale School of Medicine Yale University New Haven Connecticut USA

**Keywords:** altruism, depression, dictator game, internalizing symptoms, parental mentalising, parental reflective functioning, prosocial behaviour

## Abstract

Mental health has a profound impact on how we interact with the world. How it shapes prosocial behaviour during middle childhood, a period crucial for establishing healthy relationships, remains poorly understood. Moreover, whilst child mental health and prosocial behaviour are influenced by caregiving experience more broadly, less is known about how they are shaped by parental reflective functioning (PRF), that is parents' capacity to represent their child's underlying mental states. A longitudinal design, with assessments at baseline and 1 year follow‐up, was used with 233 children (111 boys; 6–13 years old; 54.9% White, 17.2% Asian, 2.58% Black, 14.2% Multiple ethnic groups, 2.58% Other, 8.58% data unavailable). Using path modelling, we examined interrelations between baseline PRF, baseline child internalizing symptoms, and follow‐up child altruistic prosocial behaviour. At baseline, PRF was associated with child internalizing symptoms, whilst PRF and internalizing symptoms positively predicted altruistic behaviour 1 year later. These findings suggest that mental health and caregiving experience are key influences on altruistic behaviour in childhood.

**Statement of Contribution**

- Extant research suggests that internalising symptoms are positively related to prosocial behaviour. To our knowledge, no study to date has investigated this association longitudinally, in relation to altruistic prosocial behaviour, as captured via behavioural economic paradigms. Moreover, whilst prosocial behaviour is impacted by caregiving experience, less is known about how it is shaped by parental reflective functioning (PRF).
- Using a sample of 6–13 year olds, the current study finds that internalising symptoms and Interest and Curiosity (a component of PRF) longitudinally predict altruistic prosocial behaviour, indexed by the Dictator Game. The current study therefore demonstrates important longitudinal associations between caregiving, mental health and social behaviour in middle childhood.

## PROSOCIAL BEHAVIOUR: DEVELOPMENT AND MEASUREMENT

Prosocial behaviour, defined as voluntary behaviour intended to benefit others (Hawley, 2014), is a fundamental tenet of functioning societies. It can manifest in different ways and includes behaviours such as sharing, helping, and comforting (Dunfield, 2014). In the context of sharing, prosocial behaviour can take the form of reciprocity (sharing in response to others behaviour), generosity (sharing regardless of others' actions), and altruism (sharing even at the cost to oneself). Such behaviour is largely considered to be evolutionarily selected (see Penner et al., 2005), and may provide a group selection advantage (Wilson, 1975). Furthermore, prosocial behaviour is associated with greater social connectedness (O'Malley et al., 2012) and promotes greater happiness (Dunn et al., 2008). Prosocial behaviour emerges after the first year of life (Schmidt & Sommerville, 2011) and undergoes marked changes across development (Fabes & Eisenberg, 1998). These fledgling prosocial tendencies subsequently increase from infancy to childhood (Zahn‐Waxler et al., 2001) and into adolescence (Fabes & Eisenberg, 1998). For instance, by 4 years of age children reject proposed resource allocations that place them at a disadvantage, while by 8 years of age children even reject proposals that put them at an advantage over others (Blake & McAuliffe, 2011). Altruistic behaviour appears to be present in 4‐year‐old children and increases with age (Benenson et al., 2007). However, whilst we have an increasing understanding of how prosocial behaviour develops across childhood, little is known about the role played by a child's mental health. Here we longitudinally examine the association between altruistic prosocial behaviour and internalizing symptoms in addition to the role of the child's caregiving experience.

Experimental investigations of prosocial behaviour in childhood can effectively draw on methodological advances in social decision‐making. Decision‐making science offers a framework to investigate the mechanisms associated with prosocial behaviour using game‐theoretic experimental paradigms (e.g. Rilling et al., 2008; Sanfey, 2007). These tasks, derived from the field of behavioural economics, are characterized by the interaction with another person, transfer of financial resources, and the possibility of real monetary reward, and have been used in both early (Benenson et al., 2007; Blake & McAuliffe, 2011; Fehr et al., 2008; House et al., 2012) and middle (Steinbeis, 2018; Steinbeis et al., 2012; Steinbeis & Over, 2017; Takagishi et al., 2015) childhood samples. Such paradigms can capture different components of prosocial behaviour. For example, in the Dictator Game, a proposer is asked to split a sum of money between themselves and another, where the amount given to the other player is considered a marker of prosocial behaviour. Specifically, behaviour in this task has been taken to reflect *altruistic* prosocial behaviour, that is, prosocial acts that benefit another even at the cost to oneself (Böckler et al., 2016). These experimental paradigms allow precision in the measurement of specific types of prosocial behaviour that in turn provide greater nuance in understanding the connection between prosocial behaviour and mental health.

## PROSOCIAL BEHAVIOUR AND INTERNALIZING SYMPTOMS

Alarcón and Forbes (2017) have proposed a positive association between internalizing symptoms and altruistic prosocial behaviour in adolescents. Specifically, they suggest that internalizing symptoms increase the degree to which adolescents are concerned with social evaluation. To reduce potential negative evaluation from others, adolescents may behave altruistically, putting others' needs before their own. Whilst not isolating *altruistic* prosocial behaviour, many studies have investigated the association between internalizing symptoms and prosocial behaviour more generally, in adolescent populations. For example, adolescents with depression and anxiety tend to cooperate more than their counterparts during social economic exchange games (McClure et al., 2007), even after co‐player betrayal (McClure‐Tone et al., 2011), while during the Ultimatum Game (a variant of the Dictator Game), adolescents with depression accept more unfair offers (Harlé et al., 2010) and propose more generous offers (Destoop et al., 2012). Collectively, it seems that, during adolescence, higher internalizing symptoms are generally associated with greater prosocial behaviour, yet there is evidence of bidirectional effects between mental health and prosocial behaviour. For example, as captured via parent or teacher report, prosocial behaviour predicts less emotional problems and internalizing symptoms in young children and adolescents (Flynn et al., 2015; Memmott‐Elison & Toseeb, 2023). However, for some, *altruistic* prosocial behaviour may serve to *increase* internalizing symptoms, due to the emotional burden of repeatedly putting others needs first, therefore creating a self‐sustaining cycle (Alarcón & Forbes, 2017) in which internalizing symptoms are maintained or worsened.

To our knowledge, no prior study has investigated the predictive association between internalizing symptoms and *altruistic* prosocial behaviour captured via game‐theoretic paradigms in the context of middle childhood. This developmental period is crucial for establishing healthy relationship patterns before adolescence, during which, peer relationships increase significantly in importance (e.g. Larson & Richards, 1991; Sebastian et al., 2010) and mental health problems are more likely to arise (e.g. Andersen & Teicher, 2008). Thus, to overcome these limitations, the present study sought to investigate the predictive relation between internalizing symptoms and altruistic prosocial behaviour during middle childhood using a longitudinal design. Here, we isolated altruistic prosocial behaviour using the Dictator Game (Böckler et al., 2016).

## PARENTAL REFLECTIVE FUNCTIONING

In addition to shedding light on the link between internalizing symptoms and altruistic behaviour, we further sought to investigate the role played by children's caregiving experience. Whilst parenting is associated with both child internalizing symptoms (e.g. Pinquart, 2017) and prosocial behaviour (e.g. Bevilacqua et al., 2021; Padilla‐Walker et al., 2016), less is known about the specific role of Parental Reflective Functioning (PRF). PRF refers to the cognitive‐affective function (Luyten et al., 2020) through which parents represent their child's mental states (such as desires, perspectives, and emotions), and their own mental states in relation to their child (Luyten, Nijssens, et al., 2017; Slade, 2005), and is related to parenting behaviours such as distress tolerance (Rutherford et al., 2015), autonomy support (Decarli et al., 2023), and parental sensitivity (Stacks et al., 2014). PRF deficits can present as insufficient or lacking, whereby parents are less able or motivated to understand their child's mental states (i.e. hypomentalizing), or as excessive, such that parents make unrealistically elaborate interpretations of their child's behaviour (i.e. hypermentalizing; Luyten, Mayes, et al., 2017; Luyten, Nijssens, et al., 2017). PRF can be divided into three subcomponents, as measured by the Parental Reflective Functioning Questionnaire (PRFQ; Luyten, Mayes, et al., 2017). Interest and Curiosity reflects the degree of interest parents have about their child's mental states, whereby too low levels reflect a lack of interest in understanding their child (hypomentalising), yet too high levels may reflect excessive interest to an intrusive degree (hypermentalizing). Certainty about Mental States captures the parent's confidence in their understanding of their child; low certainty reflects a reduced capacity to understand their child's inner world (hypomentalizing), yet excessively high levels of certainty may reflect a reduced appreciation of the opacity of mental states (hypermentalizing). Finally, Prementalising Modes reflects a reduced capacity to accurately enter the inner world of the child and is often characterized by the attribution of malevolent intentions to their behaviour (hypermentalizing; Luyten, Nijssens, et al., 2017).

## PARENTAL REFLECTIVE FUNCTIONING, INTERNALIZING SYMPTOMS, AND PROSOCIAL BEHAVIOUR

PRF may help facilitate altruistic behaviour in children, by building their own reflective functioning capacity (Luyten, Nijssens, et al., 2017; Slade, 2005). Specifically, by displaying an interest in, and capacity to correctly interpret, children's underlying mental states, children develop not only an appreciation of the separateness of others' thoughts, feelings, and needs to their own but also the capacity to correctly identify them in others (Slade, 2005). Indeed, environments characterized by good PRF can facilitate the capacity to cognitively or affectively represent others' mental states in children and adolescents (Benbassat & Priel, 2012; Lee et al., 2022; Nijssens et al., 2021), and such a capacity is likely to have downstream consequences for altruistic behaviour; by being more able to understand other's needs, or feel other's feelings, children may be more able, or motivated, to behave altruistically, putting other's needs above their own. Relatedly, we note that representing other's mental states has been linked to prosocial behaviour in 5–6‐year‐old children (Lee et al., 2022; McLoughlin & Over, 2019; Williams et al., 2014). To our knowledge, no study to date has examined the association between PRF and altruistic prosocial behaviour in children whilst utilizing game‐theoretic measures. Such an approach, we believe, offers greater precision and nuance and thus enriches our understanding of the social sequalae of PRF. Nonetheless, some studies have investigated PRF and social behaviour more generally. For example, Kapeleris (2014) found a positive association between general reflective functioning in mothers and child social skills while, Menashe‐Grinberg et al. (2022) report that improving PRF led to significant reductions in child behavioural problems, and Lee et al. (2022), report associations between PRF and prosocial behaviour in preschoolers. Further, Ghanbari et al. (2023) report linear associations between child psychosocial functioning and Prementalising Modes and Interest and Curiosity, whilst Gordo et al. (2020), report a linear association between socioemotional development and Prementalising Modes. Collectively, these findings suggest that PRF may serve as an important factor for the development of altruistic prosocial behaviour in children.

PRF may also help prevent or reduce internalizing symptoms in children, by building their emotion regulation capacities (Luyten, Nijssens, et al., 2017; Slade, 2005). Indeed, PRF can help parents represent their child's mental states (e.g. Slade, 2005), in turn facilitating affect mirroring: the process through which parents accurately reflect their child's emotional state, whilst adding elements of humour or playfulness; (e.g. Gergely & Watson, 1996). This reflected affect, known as a second‐order representation (a representation of the child's experience in the mind of their parent) can be subsequently internalized by the child and used as a basis for representing and regulating their own emotions (Fonagy & Target, 1997; Gergely & Watson, 1996; Luyten, Nijssens, et al., 2017; Slade, 2005), with emotion regulation, in turn, negatively related to child internalizing symptoms (Kim‐Spoon et al., 2013). Many studies have begun to investigate the relation between PRF and child internalizing symptoms. For example, Kapeleris (2014) find that lower general reflective functioning in mothers was linked with higher child internalizing symptom, whilst Ensink et al. (2017) find that PRF can buffer the effect of childhood maltreatment on internalizing symptoms. Finally, when using the PRFQ, Interest and Curiosity, Certainty about Mental States, and Prementalising Modes were all linearly associated with child internalizing symptoms (Khoshroo & Seyed Mousavi, 2022), suggesting that PRF may be an important factor relating to internalizing symptoms in children.

## AIMS AND HYPOTHESES

To date, extant research suggests that greater levels of PRF may predict altruistic behaviour in children, while lower levels of PRF may be associated with greater child internalizing symptoms, in turn, predicting more altruistic behaviour, possibly as a response to heightened social‐evaluative concern. The current study aimed to investigate these interrelations between PRF, internalizing symptoms, and altruistic prosocial behaviour in children, using path modelling. Using a longitudinal design, we hypothesized that baseline internalizing symptoms would predict altruistic prosocial behaviour in children at 1 year follow‐up. In relation to parenting, we explored whether PRF predicted altruistic prosocial behaviour at 1 year follow‐up. Finally, we also hypothesized that PRF would be associated with child internalizing symptoms at baseline.

## MATERIALS AND METHODS

### Design and procedure

The current study used a dataset from a longitudinal cognitive training study published elsewhere (Ganesan et al., 2024) focusing on the effects of cognitive control training on a host of cognitive and neural outcome measures. Here, we used a within‐subjects, longitudinal design, with two time points (baseline and 1 year follow‐up), whereby all variables (PRFQ, internalizing symptoms and Dictator Game) were collected at both timepoints. When data collection was interrupted by the COVID‐19 pandemic, data collection was moved online (using the research platform, Gorilla, www.gorilla.sc; Anwyl‐Irvine et al., 2020).

### Participants

A total of 262 participants were recruited from schools in Greater London, using a convenience sampling approach. Participants were aged between 6.0 and 13.3 years old (mean = 9.0, standard deviation = 1.6). Participants were excluded if they had a formal diagnosis of neurodevelopmental disorders or violated MRI safety criteria (i.e. metal in body or claustrophobia), due to the neuroimaging component of the wider study (Ganesan et al., 2024). Due to data loss or dropout, 29 participants were removed, leaving a final sample size 233 (111 boys). The ethnicity composition is as follows: 54.9% White, 17.2% Asian, 2.58% Black, 14.2% Multiple ethnic groups, 2.58% Other and 8.58% data unavailable. Socioeconomic status (SES) was calculated using both parents' education and employment (Cirino et al., 2002; Hollingshead, 1975; von Stumm et al., 2020). Parents were asked to rate their current occupation and level of education for themselves and their partner, on a scale of 1 (highest) to 5 (lowest; see Table S1). These four questions were averaged giving an overall score of SES from 1 to 5. The mean SES score (*n* = 223) for the sample was 1.64, suggesting a positive skew.

### Measures

#### Parental reflective functioning

PRF was measured using the PRFQ, an 18‐item self‐report scale that has previously been used in parents of school‐aged children (Khoshroo & Mousavi, 2022; Pazzagli et al., 2018). Participants respond to items using a Likert scale from 1 (*strongly disagree*) to 7 (*strongly agree*). Items are averaged into the three subscales: Prementalizing Modes, including items such as “My child cries around strangers to embarrass me”, Certainty about Mental States, including items such as “I can completely read my child's mind”, and Interest and Curiosity including items such as “I like to think about the reasons behind the way my child behaves and feels”. The subscales show good concurrent validity (Ghanbari et al., 2023) and internal consistency (Luyten, Mayes, et al., 2017). The Prementalising Modes subscale displayed poor internal consistency with alpha values of .374 (6 items; *n* = 233), yet the Certainty about Mental States (6 items; *n* = 233) and Interest and Curiosity (6 items; *n* = 233) subscales displayed good internal consistency with alpha values of .718 and .830 respectively.

#### Internalizing symptoms

Given that both depression and anxiety are related to prosocial behaviour profiles (McClure et al., 2007; McClure‐Tone et al., 2011; Destoop et al., 2012; Harlé et al., 2010), we computed a composite score of internalizing symptoms in keeping with transdiagnostic approaches to mental health and development. Specifically, internalizing symptoms were captured from the Generalized Anxiety Disorder subscale (GAD; example items including “Has difficulty controlling worries”) and Depression subscale (example items including “Has little confidence, feels inferior to others, or is very self‐conscious”) of the parent‐report version of the Child and Adolescent Symptom Inventory‐4R (CASI‐4R; Gadow & Sprafkin, 2005). The CASI‐4R GAD subscale has good internal consistency (Burgers & Drabick, 2016) while the Depression subscale has good incremental validity (Salcedo et al., 2018). For the Depression subscale, the seven dimensional items, in addition to the impairment item (“How often do the above behaviours interfere with your child's ability to do schoolwork or get along with others?”) were summed. For the GAD subscale, we summed all items, excluding an item also found in the ADHD subscale (“Has difficulty paying attention to tasks or activities”; Sukhodolsky et al., 2008), and Depression subscale (“Has low energy levels or is tired for no apparent reason”), and including the impairment item. The computed GAD and Depression subscales were then summed to form a composite score of internalizing symptoms. Such an approach ensures the construct is focused solely on symptoms implicated in internalizing symptoms, prevents artificial inflation of internalizing symptoms by eliminating duplicate items found in both GAD and Depression, and accounts for the degree of perceived functional impairment. The GAD and Depression subscales displayed good internal consistency with alpha values of .788 (7 items; *n* = 221) and 0.824 (8 items; *n* = 221) respectively.

#### Altruistic prosocial behaviour

Altruistic prosocial behaviour was measured using a single‐trial version of the Dictator Game. This task has been used extensively in developmental populations, including the present age range (e.g., Benenson et al., 2007; Blake & McAuliffe, 2011; Fehr et al., 2008; House et al., 2012; Steinbeis, 2018; Steinbeis et al., 2012; Steinbeis & Over, 2017; Takagishi et al., 2015) and found to associate with real charity donations in adults (Bekkers, 2007), and fairness preferences and honesty‐humility personality traits in children (Allgaier et al., 2020; Gummerum et al., 2008). During the task, participants must decide how to divide coins between themselves and their partner, with the number of coins given (0–6) indexing altruistic behaviour. Researchers verified that children understood the game and reiterated the rules using 3 questions and answers: 1. “How many coins will you start off with in this game?”, “You will have 6!”; 2. “How many coins will the other child start off with during the game?”, “They will have 0!”; 3. “What do you use the coins for?”, “A present at the end of the study!”. When the Covid‐19 pandemic occurred, data collection was moved online as prior research attests the validity of online methods when collection decision‐making data in children (Nussenbaum et al., 2020). All online data collection took place at follow‐up and therefore all children had prior interaction with researchers ensuring task understanding.

### Statistical analysis

#### Imputation

As 16.4% of data was missing, we conducted Little's test using SPSS (version 29) which revealed no evidence that data was not missing completely at random, *χ* ^2^ = 159.8(138), *p* = .098. We therefore performed Multiple Imputation by Chained Equations creating 50 datasets, with 20 iterations, using the R package MICE (Buuren & van Buuren & Groothuis‐Oudshoorn, 2011). Outliers (± 2 *SD*) were removed before imputing to prevent biased estimates. One dataset was selected at random for subsequent analysis, leading to a sample of 221 participants with complete data. Figure S1 depict the distributions of the imputed and original datasets, whilst Table S2 lists the predictor variables and estimation methods used for the imputation.

#### Preliminary analysis

To assess the initial associations between the variables of interest, we ran FDR‐corrected Pearson's correlations. Additional preliminary analysis involved examining descriptive statistics and assessing the path modelling assumption of multivariate normality (within our non‐categorical variables) using Mardia's test (Mardia, 1970).

#### Path modelling

To probe the directional organization of PRF, internalizing symptoms, and follow‐up altruistic prosocial behaviour, two competing models were built: one full model, allowing all associations between variables of interest to be estimated, and one restricted model, within which only a subset of associations between the variables of interest were estimated. Both models included the same covariates; given the study used a cognitive training dataset with a large age range, training group and age were included. Furthermore, when predicting follow‐up altruistic prosocial behaviour, both models included baseline altruistic prosocial behaviour to capture change. The full and restricted models were compared on model fit indices accounting for model complexity (AIC and BIC). The best fitting model was selected, after which, model fit was further probed with the following fit indices: the Chi square test, comparing the proposed model to a saturated model, CFI with a cutoff of 0.95 (Hu & Bentler, 1999), RMSEA with a cutoff value of 0.08 (Little, 2013), and SRMR with a cutoff value of 0.08 (Hu & Bentler, 1999). Analyses were conducted in the statistical software package R (R Team, 2021).

## RESULTS

### Preliminary analysis

Descriptive statistics, Pearson's correlation coefficients with FDR‐corrected p values, and associated sample sizes are presented in Table 1. Mardia's test revealed evidence of multivariate kurtosis (2.22, *p* = .027) and multivariate skewness (242.67, *p* < .001), suggesting that the data are not multivariate normally distributed.

**TABLE 1:** Descriptive statistics and pearson's correlation matrix.

	Prementalising (baseline)	Prementalising (follow‐up)	Interest (baseline)	Interest (follow‐up)	Certainty (baseline)	Certainty (follow‐up)	Internalizing (baseline)	Internalizing (follow‐up)	Altruism (baseline)	Altruism (follow‐up)
Prementalising (Baseline)	–									
Prementalising (Follow‐up)	.48*** (221)	–								
Interest (Baseline)	−.16* (221)	−.14 (221)	–							
Interest (Follow‐up)	−.13 (221)	−.30*** (229)	.64*** (221)	–						
Certainty (Baseline)	−.16* (221)	−.15 (221)	.04 (221)	.13 (221)	–					
Certainty (Follow‐up)	−.14 (221)	−.17* (229)	−.07 (221)	.07 (230)	.64*** (221)	–				
Internalizing (Baseline)	.13 (221)	.11 (221)	.14 (221)	.07 (221)	−.20* (221)	−.27*** (221)	–			
Internalizing (Follow‐up)	.13 (221)	.12 (228)	.11 (221)	.13 (229)	−.20* (221)	−.39*** (229)	.57*** (221)	–		
Altruism (Baseline)	.03 (221)	.03 (228)	.13 (221)	.01 (229)	−.05 (221)	.01 (229)	−.02 (221)	−.09 (228)	–	
Altruism (Follow‐up)	.07 (221)	.04 (229)	.24** (221)	.15 (230)	−.07 (221)	−.06 (230)	.16* (221)	.11 (229)	.19* (229)	–
Mean	1.64	1.62	5.78	5.76	4.39	4.41	5.31	5.58	1.83	2.15
SD	0.55	0.55	0.82	0.83	1.18	1.18	4.31	4.31	1.27	1.27

*Note*: FDR‐corrected Significance Thresholds: *** = *p* < .001, ** = *p* < .01, * = *p* < .05. Brackets indicate sample size.

Abbreviation: *SD*, Standard Deviation.

### Path modelling

As the multivariate normality assumption was violated, modelling was conducted using robust maximum likelihood estimations, which have been shown to be robust against violations in multivariate normality (Zhong & Yuan, 2011), using the R package Lavaan (Rosseel, 2012). As only one of the three baseline PRF components (Interest and Curiosity) was significantly correlated with follow‐up altruistic prosocial behaviour, in contrast to the full model (allowing associations between all variables), the restricted model did not estimate the direct effects of Prementalising Modes or Certainty about Mental States on follow‐up altruistic behaviour. The restricted model (AIC = 1986, BIC = 2033) displayed superior fit when compared to the full model (AIC = 1988, BIC = 2042), after accounting for complexity. To preserve parsimony and avoid overfitting, we therefore selected the restricted model for interpretation. The restricted model provided an excellent fit of the data (Scaled *χ* ^2^ = 3.70 (3), *p* = .296; robust CFI = 0.98; robust RMSEA = 0.03, robust 90% CI = 0.000 and 0.124; SRMR = 0.02; Hu & Bentler, 1999; Little, 2013).

**FIGURE 1:**
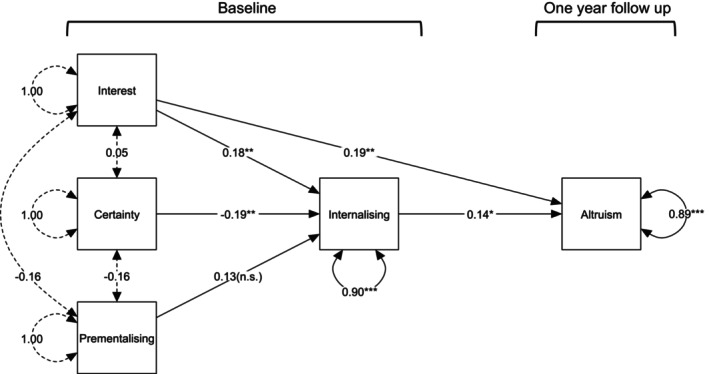
Restricted path model of PRFQ, internalizing symptoms, and altruistic prosocial behaviour. Single‐ended arrows reflect directional relationships, double‐ended, dotted arrows reflect bidirectional relationships between the three exogenous variables and circular, double‐ended arrows reflect residual variance. Edge labels are standardized path coefficients. Edge significance is denoted by * = *p* < .05, ** = *p* < .01, *** = *p* < .001, n.s. = not significant. Total sample used for path modelling = 221. Node Labels: Interest = baseline Interest and Curiosity, Certainty = baseline Certainty about Mental States, Prementalising = baseline Prementalising Modes, Internalizing = baseline internalizing symptoms, Altruism = follow‐up altruistic prosocial behaviour. Covariates include training group and age. When predicting follow‐up altruistic prosocial behaviour, baseline altruistic prosocial behaviour was added as a covariate.

As displayed in Figure 1, internalizing symptoms at baseline significantly predicted altruistic prosocial behaviour at follow‐up, such that greater levels of internalizing symptoms were predictive of greater altruistic behaviour a year later, whilst controlling for training group, age and baseline altruistic prosocial behaviour. Moreover, Interest and Curiosity at baseline significantly predicted altruistic prosocial behaviour 1 year later, such that greater levels of Interest and Curiosity predicted greater degrees of altruistic behaviour, whilst accounting for training group, age, and baseline altruistic prosocial behaviour. Finally, whilst controlling for training group and age, baseline Certainty about Mental States and Interest and Curiosity were negatively and positively associated with baseline internalizing symptoms, respectively, whilst the association between Prementalising Modes and child internalizing symptoms did not reach statistical significance. Full path modelling statistics (path coefficients, standard error, *z* statistic and *p* values) for standardized path coefficients are presented in Table 2, whilst path modelling statistics for unstandardized coefficients are displayed in Table S3. Additionally, we present the model fit indices, path diagram, and standardized and unstandardized path modelling statistics for the full model in Table S4, Figure S2 and Tables S5 and S6.

**TABLE 2:** Standardized path modelling statistics.

Relationship	Path coefficient (standardized)	*SE*	*z*	*p*
Interest and Curiosity and Internalizing Symptoms	0.18	0.06	2.90	.004
Certainty about Mental States and Internalizing Symptoms	−0.19	0.06	−3.15	.002
Prementalising Modes and Internalizing Symptoms	0.13	0.07	1.80	.072
Interest and Curiosity and Altruistic Prosocial Behaviour	0.19	0.06	3.01	.003
Internalizing Symptoms and Altruistic Prosocial Behaviour	0.14	0.07	2.12	.034

Abbreviations: *p*, statistical significance; *SE*, standard error, *z*, *z* statistic.

## DISCUSSION

The current study aimed to investigate the interrelations between PRF, internalizing symptoms, and altruistic prosocial behaviour in children. In line with our predictions, we found that baseline internalizing symptoms was predictive of altruistic prosocial behaviour in children 1 year later. Furthermore, we also found that Interest and Curiosity was predictive of altruistic prosocial behaviour 1 year later. Finally, significant associations between Interest and Curiosity and Certainty about Mental States and child internalizing symptoms emerged at baseline. These findings demonstrate critical longitudinal associations between parenting, mental health symptoms and social behaviour during childhood.

In line with our primary hypothesis, we found a significant longitudinal association between baseline internalizing symptoms and altruistic prosocial behaviour 1 year later, such that greater internalizing symptoms predicted greater degrees of altruistic behaviour. This aligns with, and extends, previous literature finding cross‐sectional relations between prosocial behaviour and internalizing symptoms (Destoop et al., 2012; Harlé et al., 2010; McClure et al., 2007; McClure‐Tone et al., 2011). One interpretation of these findings is that children with greater internalizing symptoms display greater fears of negative social evaluation, which leads to altruistic behaviour, thus helping to increase the likelihood of positive social interactions or decrease the likelihood of negative interactions (Alarcón & Forbes, 2017). Whilst for some, engaging in altruistic acts may serve to bolster resilience to internalizing symptoms (e.g. Memmott‐Elison & Toseeb, 2023), for others, entrenching a pattern of putting other's needs first may, over time, become emotionally draining, leading to further internalizing symptoms (Alarcón & Forbes, 2017). We speculate that this may in fact contribute to an increased likelihood of clinical levels of anxiety and depression in adolescence, though this requires systematic evaluation in future research.

The Interest and Curiosity component of PRF was predictive of follow‐up altruistic prosocial behaviour, whereby greater levels of baseline Interest and Curiosity predicted greater degrees of altruistic behaviour in children. This appears to be broadly consistent with literature attesting to the role of parents' general reflective functioning in child social skills (Kapeleris, 2014), psychosocial functioning (Ghanbari et al., 2023), prosocial behaviour (indexed by caring behaviour; Lee et al., 2022), and its protective role in child behavioural problems (Menashe‐Grinberg et al., 2022). One possibility is that the experience of being responded to in an open and curious manner may facilitate the capacity to cognitively or affectively represent other's mental states. Indeed, PRF is associated with general reflective functioning in adolescents (Benbassat & Priel, 2012), while Interest and Curiosity specifically is related to empathy in preschoolers (Lee et al., 2022). Empathy, in turn, appears to be associated with prosocial behaviour in 5–6‐year‐old children and preschoolers (Lee et al., 2022; McLoughlin & Over, 2019; Williams et al., 2014). Future research could consider testing this possibility in the context of a mediation model, in addition to exploring the possible moderating role of age.

Surprisingly, we did not detect a significant association between Prementalising modes and child internalizing symptoms at baseline. Whilst appearing inconsistent with prior literature (e.g. Khoshroo & Seyed Mousavi, 2022), we believe such a finding may reflect the poor internal consistency of the Prementalising Modes subscale (*α* = .374), which would have limited sensitivity and statistical power. We find a positive association between Interest and Curiosity and child internalizing symptoms at baseline, in line with Khoshroo and Seyed Mousavi (2022). Given that in the current study, and that of Khoshroo and Seyed Mousavi (2022), both PRF and child internalizing symptoms are captured by parent report, it is possible that these relations represent epiphenomena. For example, parents with greater interest in their child's mental states may also be more able to detect and report on their child's mental health problems, which therefore may be driving this positive association. However, we acknowledge that excessive degrees of Interest and Curiosity may reflect hypermentalizing and be perceived as intrusive to the child, leading to higher levels of internalizing symptoms (Khoshroo & Seyed Mousavi, 2022). We find a negative relation between Certainty about Mental States and child internalizing symptoms at baseline, partially consistent with Khoshroo and Seyed Mousavi (2022), who report a negative association between Certainty about Mental States and internalizing symptoms, yet only in preschoolers. Whilst excessive degrees of certainty can reflect hypermentalising and have deleterious effects on child development (e.g. Luyten, Nijssens, et al., 2017), the distribution of Certainty about Mental States in our current sample does not show a skew (Figure S1) and therefore higher levels seem unlikely to represent hypermentalizing, but rather moderately high degrees of certainty, which may be related to lower levels of internalizing symptoms via emotion regulation. Indeed, parents with moderately high levels of certainty may be better able to understand and reflect their children's emotional state, which children can internalize and use to regulate their emotions (e.g. Fonagy & Target, 1997). Whilst we cannot rule out the possibility of this relation also reflecting an epiphenomenon, we believe this is less likely due to the direction of the association. If this relation emerged only because parents with high degrees of certainty are more able to report on their child's mental health, then we believe the associations would more likely be positive rather than negative. Nonetheless, future research could disentangle these possibilities by examining PRF with child‐report or teacher‐report internalizing symptoms. Finally, given the cross‐sectional nature of the relations between Interest and Curiosity or Certainty about Mental States and child internalizing symptoms, the directional relations could not be discerned.

The current findings have several implications. Firstly, the longitudinal association between internalizing symptoms and altruistic behaviour lays the foundation for future research to investigate the consequences of responding to internalizing symptoms by putting others needs first: could such behaviour serve an adaptive function, leading to a reduction in internalizing symptoms, or could it lead to an increase in internalizing symptoms, in a self‐reinforcing cycle (Alarcón & Forbes, 2017)? Such research may help explicate possible developmental cascades relating to mental health problems and offer mechanistic insights into intervention approaches, such as assertiveness training or social skills interventions, each of which having shown to reduce internalizing symptoms or promote well‐being in adolescents (Parray, 2017; Reed, 1994). Secondly, our findings contribute to a growing body of literature attesting to the role of PRF in child internalizing symptoms as a possible point of intervention. Indeed, several studies have trained PRF generally and demonstrated beneficial outcomes for both parent and child (Midgley et al., 2021), including child emotion regulation (Halfon & Bulut, 2019). Whilst we find relations between Interest and Curiosity and Certainty about Mental States with child internalizing symptoms, we suggest more research should be conducted to rule out the possibilities of these associations simply emerging as an epiphenomenon (especially in the case of Interest and Curiosity) or due to the reverse association, and to determine the optimal point of Interest and Curiosity and Certainty about Mental States for child development.

The current study has several strengths. For example, our sample size, while moderate compared to other developmental cohort studies, is to our knowledge one of the largest to employ a longitudinal study design. Further, the use of game‐theoretic measures allowed for a precise isolation of prosocial behaviour, thus reducing potential confounds. Additionally, to our knowledge, this was the first longitudinal study to predict experimentally isolated altruistic prosocial behaviour, from internalizing symptoms and Interest and Curiosity, in a sample of children. However, the study was also characterized by several limitations. Firstly, the current sample was skewed in both SES and race, with the majority of participants being White with higher levels of SES, possibly limiting the generalizability of the current findings. Secondly, the use of single‐trial prosocial behaviour tasks may limit the reliability, in comparison to multi‐round versions. Furthermore, the sole use of parent‐report child psychopathology measures may be less valid than using data from multiple informants (i.e. child‐report and teacher‐report). Finally, given the cross‐sectional nature of the associations between PRF and child internalizing symptoms in the path model, the directional relations between these variables could not be discerned.

## CONCLUSION

The current study sought to examine, for the first time, the interrelations between PRF, internalizing symptoms, and altruistic prosocial behaviour in children. Children's follow‐up altruistic behaviour was predicted both by baseline levels of internalizing symptoms and their parenting experience, indexed by Interest and Curiosity. This demonstrates an important longitudinal association between parenting, mental health, and social behaviour during childhood, and thus lay the foundations for further research to explicate the developmental interrelations between prosocial behaviour and mental health as children enter into adolescence.

## AUTHOR CONTRIBUTIONS

**Daniel McGlade:** Conceptualization; writing – original draft; writing – review and editing; formal analysis; data curation. **Helena Rutherford:** Supervision; conceptualization; writing – review and editing. **Eamon McCrory:** Supervision; conceptualization; methodology; writing – review and editing. **Nikolaus Steinbeis:** Supervision; conceptualization; methodology; funding acquisition; writing – review and editing; investigation.

## ETHICS STATEMENT

Informed consent was received from parents whilst assent was received from the child. The project was approved by the UCL ethics committee (Protocol number: 12271/001).

## Supporting information


Data S1.

## Data Availability

Research data are not shared.
