# Supplementary material for: Parental reflective functioning and internalizing symptoms predict altruistic prosocial behaviour in children
Source: Br J Dev Psychol. 2025 Feb 17;43(3):755–70. doi: 10.1111/bjdp.12551 (PMC12351215; doi:10.1111/bjdp.12551)
Supplement: Supplementary file 1 — Data S1. [file BJDP-43-755-s001.docx]

**Supporting Information**

**Supporting Table S1**

***Socioeconomic Status Scoring Criteria***

|  | **Education** | **Employment** |
| --- | --- | --- |
| **1** | Postgraduate (MSc, MA, PhD) or professional qualification (e.g. law or accountancy training. | Managerial and professional occupations  (e.g. company director, head of HR, lawyer, dentist) |
| **2** | Undergraduate (BA, BSc) or equivalent (HND/HNC, City and Guilds Qualification, NVQ level 4) | Intermediate occupations  (e.g. police officer, administrative assistant, travel consultant) |
| **3** | A-level, AS-levels, NVQ level 3, BTEC diplomas | Small employers and own account workers  (e.g. interior designer, garden designer, baker) |
| **4** | GCSES, CSEs, O-levels, NVQ levels 1 & 2 | Lower supervisory and technical occupations  (e.g. interior design assistant, finance officer) |
| **5** | No formal qualifications | Semi-routine and routine occupations  (e.g. yoga teacher, leather crafter, bookseller) |

**Supporting Figure S1**

***Multiple Imputation Distribution Plots***


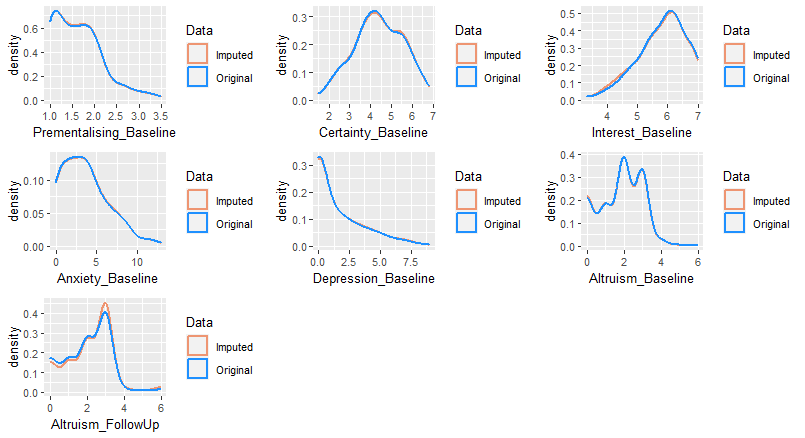


**Supporting Table S2**

***Imputation Predictor Variables and Estimation Methods***

| **Predictor Variable** | **Estimation Method (if applicable)** |
| --- | --- |
| Gender | n/a |
| Experimental Group | n/a |
| Age | n/a |
| Ultimatum Game: Proposer (baseline) | Predictive Mean Matching |
| PRFQ Subscale: Certainty about Mental States (baseline and follow up) | Predictive Mean Matching |
| PRFQ Subscale: Prementalising Modes (baseline and follow up) | Predictive Mean Matching |
| PRFQ Subscale: Interest and Curiosity (baseline and follow up) | Predictive Mean Matching |
| CASI: GAD (baseline and follow up) | Predictive Mean Matching |
| CASI: Depression1 (sum of the seven dimensional items and impairment item. Baseline and follow up) | Predictive Mean Matching |
| CASI: Depression2 (sum of the seven dichotomous items. Baseline and follow up) | Predictive Mean Matching |
| Dictator Game (baseline and follow up) | Predictive Mean Matching |
| Ultimatum Game: Responder (baseline and follow up) | Logistic Regression |
| COVID (binary. Baseline) | n/a |

**Supporting Table S3**

***Unstandardised Path Modeling Statistics: Restricted Path Model***

| **Relationship** | **Path Coefficient (Unstandardised)** | **SE** | **z** | **p** |
| --- | --- | --- | --- | --- |
| **Interest and Curiosity and Internalising Symptoms** | 0.94 | 0.34 | 2.78 | .005 |
| **Certainty about Mental States and Internalising Symptoms** | -0.70 | 0.23 | -3.03 | .002 |
| **Prementalising Modes and Internalising Symptoms** | 1.00 | 0.57 | 1.76 | .078 |
| **Interest and Curiosity and Altruistic Prosocial Behaviour** | 0.29 | 0.10 | 2.88 | .004 |
| **Internalising Symptoms and Altruistic Prosocial Behaviour** | 0.04 | 0.02 | 2.08 | .037 |

*Note.* SE= standard error, z = z statistic, p = statistical significance

**Supporting Table S4**

***Model Fit Indices: Full Path Model***

| **Model Fit Index** | **Statistic(s)** |
| --- | --- |
| Scaled Chi Square | Scaled χ² = 1.89 (1), p = .170 |
| Robust RMSEA | 0.07 (90% CI = 0.000, 0.209) |
| Robust CFI | 0.976 |
| SRMR | 0.013 |

**Supporting Figure S2**

***Full* *Path Model of PRFQ, Internalising Symptoms and Altruistic Prosocial Behaviour***

*Note***.** Single-ended arrows reflect directional relationships, double-ended, dotted arrows reflect bidirectional relationships between the three exogenous variables and circular, double-ended arrows reflect residual variance. Edge labels are standardised path coefficients. Edge significance is denoted by * = p<.05, ** = p<.01, *** = p<.001, n.s. = not significant. Total sample used for path modeling = 221. Node Labels: Interest = baseline Interest and Curiosity, Certainty = baseline Certainty about Mental States, Prementalising = baseline Prementalising Modes, Internalising = baseline internalising symptoms, Altruism = follow up altruistic prosocial behaviour. Covariates include training group and age. When predicting follow up altruistic prosocial behaviour, baseline altruistic prosocial behaviour was added as a covariate.


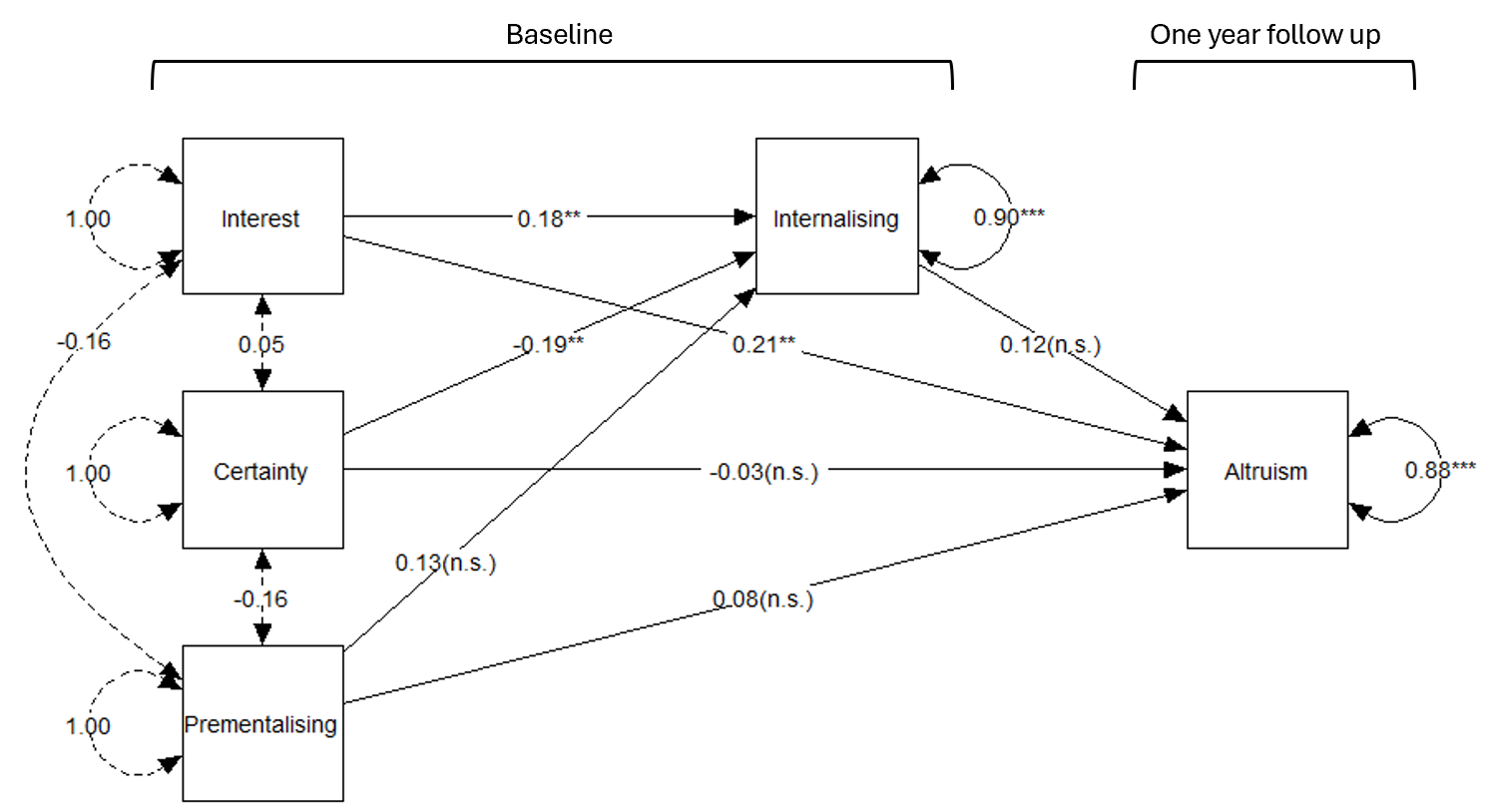


**Supporting Table S5**

***Standardised Path Modeling Statistics: Full Path Model***

| **Relationship** | **Path Coefficient (standardised)** | **SE** | **z** | **p** |
| --- | --- | --- | --- | --- |
| **Interest and Curiosity and Internalising Symptoms** | 0.18 | 0.06 | 2.90 | .004 |
| **Certainty about Mental States and Internalising Symptoms** | -0.19 | 0.06 | -3.15 | .002 |
| **Prementalising Modes and Internalising Symptoms** | 0.13 | 0.07 | 1.80 | .072 |
| **Interest and Curiosity and Altruistic Prosocial Behaviour** | 0.21 | 0.06 | 3.33 | .001 |
| **Certainty about Mental States and Altruistic Prosocial Behaviour** | -0.03 | 0.07 | -0.48 | .635 |
| **Prementalising Modes and Altruistic Prosocial Behaviour** | 0.08 | 0.07 | 1.20 | .231 |
| **Internalising Symptoms and Altruistic Prosocial Behaviour** | 0.12 | 0.07 | 1.76 | .079 |

*Note.* SE= standard error, z = z statistic, p = statistical significance

**Supporting Table S6**

***Unstandardised Path Modeling Statistics: Full Path Model***

| **Relationship** | **Path Coefficient (Unstandardised)** | **SE** | **z** | **p** |
| --- | --- | --- | --- | --- |
| **Interest and Curiosity and Internalising Symptoms** | 0.94 | 0.34 | 2.78 | .005 |
| **Certainty about Mental States and Internalising Symptoms** | -0.70 | 0.23 | -3.03 | .002 |
| **Prementalising Modes and Internalising Symptoms** | 1.00 | 0.57 | 1.76 | .078 |
| **Interest and Curiosity and Altruistic Prosocial Behaviour** | 0.32 | 0.10 | 3.18 | .001 |
| **Certainty about Mental States and Altruistic Prosocial Behaviour** | -0.03 | 0.07 | -0.48 | .634 |
| **Prementalising Modes and Altruistic Prosocial Behaviour** | 0.18 | 0.15 | 1.21 | .230 |
| **Internalising Symptoms and Altruistic Prosocial Behaviour** | 0.04 | 0.02 | 1.72 | .084 |

*Note.* SE= standard error, z = z statistic, p = statistical significance
